# Robot End Effector Tracking Using Predictive Multisensory Integration

**DOI:** 10.3389/fnbot.2018.00066

**Published:** 2018-10-16

**Authors:** Lakshitha P. Wijesinghe, Jochen Triesch, Bertram E. Shi

**Affiliations:** ^1^Department of Electronic and Computer Engineering, Hong Kong University of Science and Technology, Kowloon, Hong Kong; ^2^Frankfurt Institute for Advanced Studies, Frankfurt am Main, Germany

**Keywords:** active efficient coding, developmental robotics, sensorimotor prediction, generative adaptive subspace self-organizing map, reinforcement learning

## Abstract

We propose a biologically inspired model that enables a humanoid robot to learn how to track its end effector by integrating visual and proprioceptive cues as it interacts with the environment. A key novel feature of this model is the incorporation of sensorimotor prediction, where the robot predicts the sensory consequences of its current body motion as measured by proprioceptive feedback. The robot develops the ability to perform smooth pursuit-like eye movements to track its hand, both in the presence and absence of visual input, and to track exteroceptive visual motions. Our framework makes a number of advances over past work. First, our model does not require a fiducial marker to indicate the robot hand explicitly. Second, it does not require the forward kinematics of the robot arm to be known. Third, it does not depend upon pre-defined visual feature descriptors. These are learned during interaction with the environment. We demonstrate that the use of prediction in multisensory integration enables the agent to incorporate the information from proprioceptive and visual cues better. The proposed model has properties that are qualitatively similar to the characteristics of human eye-hand coordination.

## Introduction

To perform complex manipulation tasks, conventional robotic systems require precise calibration, which must be repeated when their physical configuration changes. In contrast, humans learn manipulation skills autonomously, and automatically recalibrate in response to physical configuration changes, e.g., due to growth and injury. Eye-hand coordination is a key skill required for these tasks. It requires the integration of multiple sensory modalities, such as vision and proprioception. Human infants appear to learn to develop a sense of themselves through observing the temporal contingency and spatial congruency of the sensory (e.g., visual, auditory, and proprioceptive) feedback received during self-produced motion, such as motor babbling (Rochat, 1998). One goal of cognitive developmental robots is to endow robots with this capability so that they will not require any manual calibration before acting in a new environment (Asada et al., 2009).

The mismatch between the motion of objects in the environment and the eye's rotational velocity creates retinal slip. During tracking motions, such as smooth pursuit, the brain attempts to minimize this slip by adapting the eye rotational velocity. Motion in the environment is generated by either self-motion (e.g., of the hand) or exteroceptive motion. When the hand moves, its motion can be sensed via two sensory modalities: retinal slip caused by the relative motion between the hand and eye and proprioceptive sensing of the position and movement of the arm. In contrast, an external object moving in the environment only generates a retinal slip. Moreover, hand motion in total darkness only provides proprioceptive information to the brain. In such conditions, the human brain has the ability to generate eye movements to follow the hand or an external target using smooth pursuit like eye movements.

In this paper, we propose a novel predictive model for learning robotic visuomotor control. The proposed system model is inspired by recent findings that neurons in the primary visual cortex (area V1) are driven not only by visual but also by the motor input. Activity in V1 was long believed to be driven only by visual inputs. However, recent findings on visual perception in awake mice have suggested that this is not true. For example, the responses in V1 depend on behavioral state (Niell and Stryker, 2011). Experiments conducted in darkness revealed that motor activity alone could trigger responses in V1 neurons (Saleem et al., 2013). The development of V1 depends upon visuomotor coupling (Attinger et al., 2017). Most relevant to this work is the discovery of cells that respond to the mismatch between the actual and predicted visual flow (Keller et al., 2012; Zmarz and Keller, 2016). This suggests that visual areas predict the sensory consequences of motor actions.

Our model takes in visual input from a camera and proprioceptive inputs from the encoders of the robot arm, and produces eye motor actions to track the moving robot hand. The model is based upon the hypothesis that the brain utilizes proprioceptive inputs to predict the visual consequences of motor actions. In line with other work in predictive coding, we use the term “predict” to refer to the process of generating an estimate of one sensory input from the values of other inputs, which may occur at the same time, rather than a more strict definition where future values are estimated from past and present values. The prediction is often used to generate a mismatch signal by comparison with the actual input. For example, Srinivasan et al. (1982) explain center-surround antagonism in the retina using predictive coding, where the predicted intensity at the center based on the surround is subtracted from the actual center signal. Rao and Ballard (1999) predict lower level cortical outputs from higher level cortical outputs. Zmarz and Keller (2016) find mismatch neurons that respond to the difference between the actual visual flow and the prediction of visual flow from self-motion. Our model is most similar to the latter work, where prediction is across sensory modalities.

There are several important novel attributes of the learning methodology compared to similar work in the literature. First, the learning does not depend on any fiducial visual marker to identify the end effector of the robot. Second, the model does not require the forward kinematics of the arm to be known. Third, pre-defined visual feature descriptors are not required, but rather are learned. Finally, our experimental results with this model suggest that the use of prediction enables the model to better integrate proprioception and vision.

The rest of the paper is organized as follows. In section Related Work, we place our work into the context of past work. Section Materials and Methods describes the model, experimental setup and learning algorithms. Then in section Results, we present experimental results comparing the tracking performance. We also compare our model characteristics with human psychophysical data. Finally, in section Discussion, we further discuss the results presenting the corresponding conclusions.

## Related work

The problem of learning end effector tracking is a part of the larger problem of autonomous learning of the body schema. The body schema is a sensorimotor representation of the body that can be used to direct motion and actions. It integrates multiple cues, including proprioception, vision, audition, vestibular cues, tactile cues, and motor cues, to represent the relations between the spatial positions of the body parts. Knowledge of the body schema can be used in a number of different tasks, e.g., end effector tracking, reaching, posture control and locomotion.

The review by Hoffmann et al. (2010) classifies body schema representations used in robotics into two classes: explicit and implicit. Both have been used to address the problem of end effector tracking. In the explicit approach (e.g., Bennett et al., 1991; Hollerbach and Wampler, 1996; Gatla et al., 2007), transformations between sensory and motor coordinates are broken down into a chain of closed form transformations where each link corresponds explicitly to part of the robot structure. The work we present here falls into the class of implicit models, where an implicit representation (e.g., a look up table or neural network) is used.

Past work has often used a point representation of the end effector, where artificial markers (e.g., color blobs) have been used to enable easy identification of the end effector (Hersch et al., 2008; Sturm et al., 2009). For example, a biologically inspired model to learn visuomotor coordination for the robot Nao was proposed in Schillaci et al. (2014). Learning occurred during motor babbling, which is similar to how infants may learn early eye-hand coordination skills. The proposed method used two Dynamic Self Organizing Maps (DSOMs) to represent the arm and neck position of the robot. The connections between the DSOMs were strengthened if the robot was looking at a fiducial marker positioned on the end effector. After learning, the robot had the ability to track the end effector by controlling the neck joints. One advantage of this model is that the method has no assumption that the forward arm kinematics of the robot is known. However, one limitation of the approach is that it required a fiducial marker.

Subsequent work has relaxed the assumption that the end effector is a point and removed the requirements for explicit markers. However, it has still required hard-coded visual feature descriptors. For example, an algorithm to learn the mapping from arm joint space to the corresponding region in image space containing the end effector was proposed in Zhou and Shi (2016), based on a measure of visual consistency defined using SIFT features (Lowe, 2004). This algorithm did not require prior knowledge of the arm model, and was robust to changes in the appearance of the end effector. Other marker-less approaches have relied upon knowledge of a 3D CAD model of the end effector (Vicente et al., 2016; Fantacci et al., 2017). Vicente et al. (2016) eliminated calibration errors using a particle filter. The likelihood associated with each particle was evaluated by comparing the outputs of Canny edge detectors applied to both the real and simulated camera images. Fantacci et al. (2017) extended this particle filter and 3D CAD model based approach to estimate the end effector pose. The likelihood was evaluated using a Histogram of Oriented Gradient (HOG) (Dalal and Triggs, 2005) based transformation to compare the two images. The approach to bootstrap a kinematic model of a robot arm proposed in Broun et al. (2014) does not require a priori knowledge of a CAD model, as it constructs a model of the end-effector on the fly from Kinect point cloud data. However, it still requires a hard-coded optical flow extraction stage to identify the arm in the image through visuomotor correlation.

Some of the limitations in the aforementioned research (e.g., the requirement for a marker and/or hard-coded image features) were addressed in our prior work (Wijesinghe et al., 2017), which proposed a multisensory neural network that combined visual and proprioceptive modalities to track a robot arm. Retinal slip during the motion was represented by encoding two temporally consecutive image frames using a sparse coding algorithm where the basis vectors were learned online (Zhang et al., 2014). The sparse coefficients were combined with proprioceptive input to control the eye to track the arm. This paper extends our previous idea by introducing a new model following the hypothesis that the brain generates internal predictions for consequences of actions.

## Materials and methods

Our approach is based on the Active Efficient Coding (AEC) framework (Zhao et al., 2012; Teulière et al., 2015), a generalization of the efficient coding hypothesis to active perception. Under the efficient coding hypothesis, the sensory data is encoded efficiently by exploiting redundancies in the statistics of the sensory input signals. In AEC, movements of the sensory organs are also learned so that the inputs can be coded efficiently. In the proposed model, visual, and proprioceptive stimuli are jointly encoded. This perceptual representation is used to generate eye movements for tracking the robot arm. Simulation of the model is performed using the iCub humanoid robot simulator, an open source robot simulator for the iCub robot (Tikhanoff et al., 2008). We provide more detail in the following subsections.

### Model architecture

**Figure 1:**
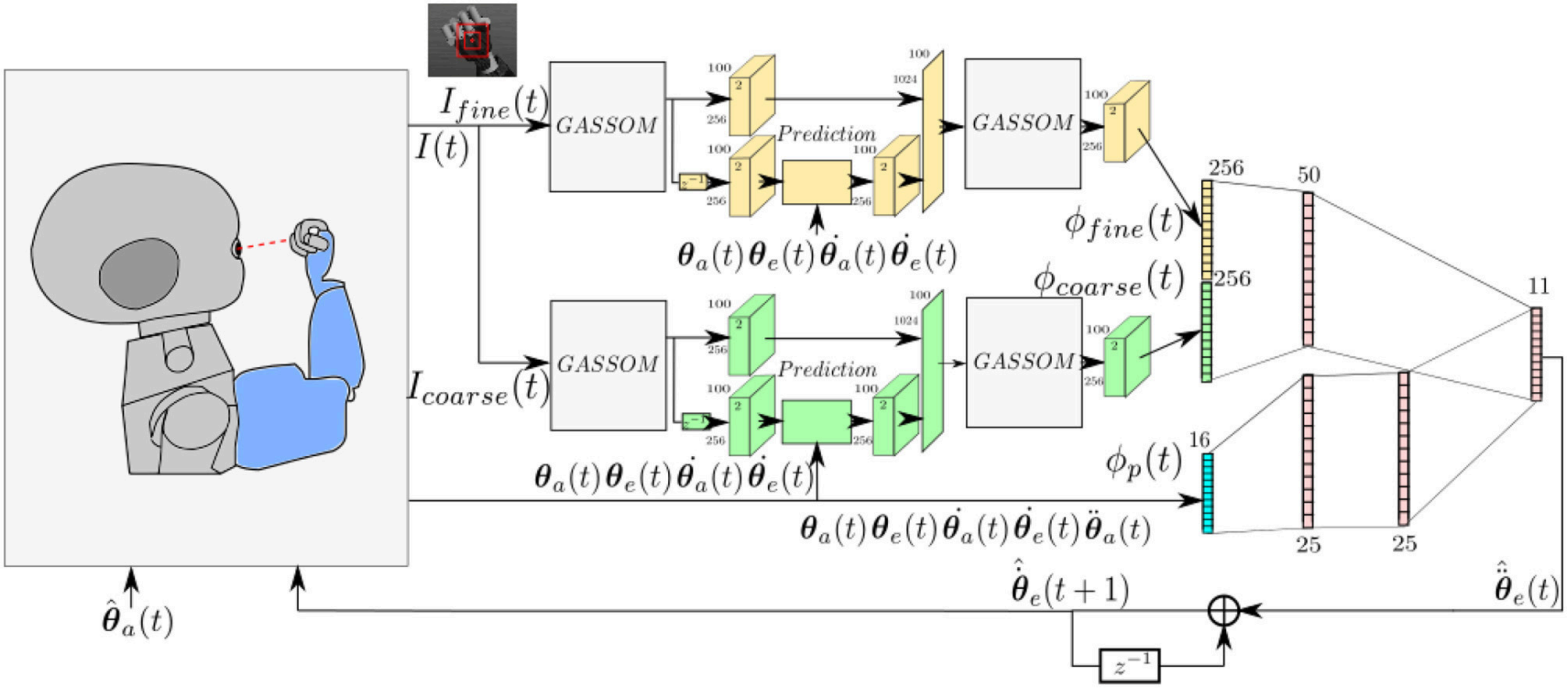
The model takes as input images at two scales and proprioceptive readings from the encoders. The eye controller represented by the neural network maps the perceptual representation to motor actions.

Figure 1 illustrates the architecture of the proposed model, which evolves in discrete time. We assume that each iteration corresponds to 40 ms.

At each iteration, the right eye of the iCub captures an image with 320 × 240 pixel resolution. Two foveal subwindows are extracted from the center of this image: a smaller 55 × 55 pixel fine scale image, I_fine_(*t*), and a larger 110 × 110 pixels coarse scale subwindow, I_coarse_(*t*), which is subsampled horizontally and vertically by a factor of two. These subwindows cover 11^*o*^ and 22^*o*^, respectively, horizontally and vertically.

The visual stimuli are encoded using Generative Adaptive Subspace Self Organizing Maps (GASSOMs) (Chandrapala and Shi, 2015). Proprioceptive inputs include the arm position, velocity, and acceleration $\left(\theta_{\text{a}}\left(t\right)\in {\mathbb{R}}^{4},{\dot{\theta }}_{\text{a}}\left(t\right)\in {\mathbb{R}}^{4},{\ddot{\theta }}_{\text{a}}\left(t\right)\in {\mathbb{R}}^{4}\right)$ and the eye position and eye velocity $\left(\theta_{\text{e}}\left(t\right)\in {\mathbb{R}}^{2},{\dot{\theta }}_{\text{e}}\left(t\right)\in {\mathbb{R}}^{2}\right)$ as reported by the motor encoders. The prediction module predicts the sensory consequences of the arm and eye motions based on proprioceptive inputs. This enables the model to reduce the correlation between the visual features and the proprioceptive inputs during self-motion. Finally, the visual and proprioceptive inputs are integrated using an Artificial Neural Network (ANN) to generate pan and tilt eye acceleration commands, ${\ddot{\hat{\theta }}}_{\text{e}}\left(t\right)\in {\mathbb{R}}^{2}\;$, enabling the right eye to track the robot arm. The “∧” symbol is added to distinguish motor commands from proprioceptive information.

Given the eye acceleration command, the eye velocity is obtained by;

(1) $$\begin{array}{l} {\dot{\hat{\theta }}}_{\text{e}}\left(t+1\right)={\dot{\hat{\theta }}}_{\text{e}}\left(t\right)+{\ddot{\hat{\theta }}}_{\text{e}}\left(t\right). \end{array}$$

Equation (1) is similar to the model for the maintenance of pursuit described in Lisberger (2010), where an efference copy of the eye velocity command is fed back in order to determine the current command for eye velocity in the immediate future. This enables eye velocity to be maintained automatically. In our model, both image motion and arm motion can drive changes in eye velocity through the eye acceleration command.

The model presented here uses only afferent information to determine the eye acceleration. In biological systems, both afferent and efferent signals from the arm are involved in arm-eye coordination control. For deafferented monkeys, smooth pursuit eye movements disappeared while tracking a target moved by active arm movements in darkness (Gauthier and Mussa Ivaldi, 1988). Steinbach (1969) found differences in ocular tracking of active and passive hand motions, which suggest that efference commands also play a crucial role. Gauthier and Mussa Ivaldi (1988) and Gauthier et al. (1988) suggested that efferent signals serve to synchronize the onsets of arm and eye motions, whereas proprioceptive signals serve to couple the eye and hand motor signals once movement has started. Subsequent experiments have provided additional support for this hypothesis (Vercher et al., 1996). Since we use only afferent information, the model may provide an account for differences in performance once movement has started. We leave the integration of an efference copy to future extensions of the model.

During training, we control four degrees of freedom (DoF) among the seven DoF in the iCub robot arm. The three joints in the wrist are fixed, and the remaining four joints (the shoulder pitch, shoulder roll, shoulder yaw, and the elbow joint) are controlled. We fix the wrist angles so that the palm of the iCub robot remains approximately parallel to the image plane. As described below, one assumption of our approach is that the retinal flow is uniform across both fine and coarse foveal image regions. Keeping the wrist angle fixed ensures that the image of the palm covers the foveal images when the gaze vector intersects the center of the palm. Modifying the algorithm so that the image region used to generate motor commands vary in size automatically may enable the algorithm to allow all DoF to vary. During testing, we allow the wrist to move (see section Qualitative Evaluation of Performance).

In the following, we describe the model components in more detail.

### Visual and proprioceptive features

The model encodes the visual stimuli in two stages. In the first stage, each foveal image is divided into a 10 × 10 array of 10 × 10 pixel overlapping patches, ${\text{x}}_{1,s,i}\left(t\right)\in {\mathbb{R}}^{100}$, with a stride of 5 pixels, where *s* indexes scale and *i* ∈ {1, 2, …, *P*} indexes the patch (*P* = 100). The subscript “1” indicates the stage.

Patches are encoded using GASSOMs (Chandrapala and Shi, 2015). The GASSOM is a probabilistic generative extension of the Adaptive Subspace Self Organizing Map (ASSOM) (Kohonen, 1996). It assumes that each input vector **x**_1,*s,i*_(*t*) is generated by one of *N* = 256 nodes. The generating nodes evolve according to a first order Markov process. Each node is associated with a two dimensional subspace spanned by basis vectors specified by the orthogonal columns of the matrix ${\text{B}}_{1,s,n}\in {\mathbb{R}}^{100\times 2}$. The input is generated by the node by choosing a Gaussian distributed vector lying on the subspace plus a small noise vector lying in the orthogonal complement of the subspace. Using the algorithm described in Chandrapala and Shi (2015), both the transition matrix of the Markov process and the node subspaces are learned so as to maximize the likelihood of the input sensory data. The learned transition matrices have high self-transition probability, which implies that the node generating input *t*−1 is likely to generate the input at time *t*, a property we refer to as “slowness.”

The output of the GASSOM is the set of projections of the input vector onto the *N* subspaces.

(2) $$\begin{array}{l} {\;\text{p}}_{1,s,n,i}\left(t\right)={\text{B}}_{1,s,n}^{T}{\;\text{x}}_{1,s,i}\left(t\right). \end{array}$$

When the eye is viewing the end effector, these projections change in a regular manner, which depends upon the movement of the arm and the eye.

**Figure 2:**
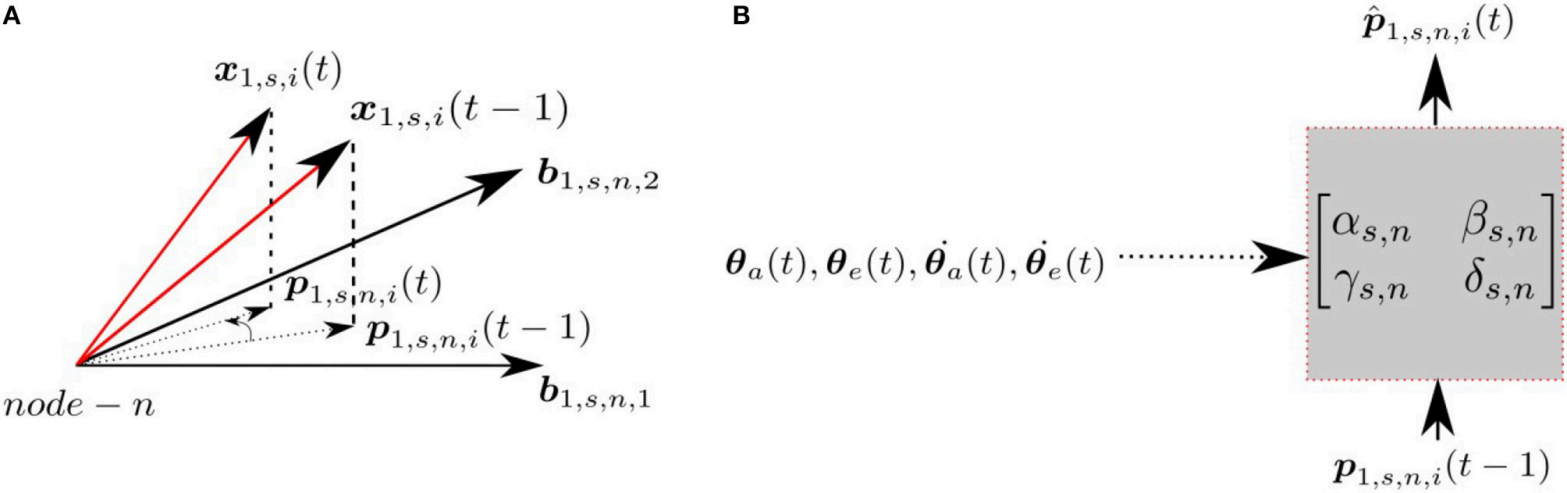
**(A)** Projections at time **t** and **t − 1** for a given node **n**, scale **s** and patch **i**. **(B)** The transformation matrix projecting current projection at time **t**, given the projection at time **t − 1** with the proprioceptive input.

Each node at each scale has an associated prediction module, which predicts the projection of the input at time *t*, **p**_1,*s,n,i*_(*t*), given the input at time *t*−1, **p**_1,*s,n,i*_(*t*−1) and the proprioceptive signals encoding the arm/eye position/velocity ($\theta_{\text{a}}\left(t\right),{\dot{\theta }}_{\text{a}}\left(t\right),\theta_{\text{e}}\left(t\right),{\dot{\theta }}_{\text{e}}\left(t\right)$). Figure 2 indicates the projections of the patch **x**_1,*s,i*_ at times *t* and *t*−1 and the corresponding transformation in the subspace. The prediction module assumes the transformation can be modeled as a linear mapping, where the predicted projection at time *t* is given by;

(3) $$\begin{array}{l} {\;\hat{\text{p}}}_{1,s,n,i}\left(t\right)=\left[\begin{array}{cc} \alpha_{s,n} & \beta_{s,n}\\ \gamma_{s,n} & \delta_{s,n} \end{array}\right]{\;\text{p}}_{1,s,n,i}\left(t-1\right), \end{array}$$

where the α, β, γ and δ parameters for each scale *s* and node *n* depend upon the arm/eye position/velocity. These parameters are computed using a neural network with four inputs ($\theta_{\text{a}}\left(t\right),{\dot{\theta }}_{\text{a}}\left(t\right),\theta_{\text{e}}\left(t\right),{\dot{\theta }}_{\text{e}}\left(t\right)$), one hidden layer containing 25 hidden units with tanh activations, and four linear output neurons. Since all patches share the same α, β, γ, and δ parameters, we are assuming that the retinal flow is uniform across the foveal images. By performing the prediction in the projected subspace, rather than the original high dimensional pixel space, we simplify the task of prediction.

The parameters of the 512 (2 scales × 256 nodes) neural networks are learned online using stochastic gradient descent, where the weights are updated every iteration. Since each foveal image contains 100 patches, we average the gradients of the prediction error across the 100 patches, and update the weights of each neural network with the average gradient.

The second GASSOM encodes the concatenated vectors **p**_1,*s,n,i*_(*t*) and ${\;\hat{\text{p}}}_{1,s,n,i}\left(t\right)$ corresponding to all the nodes *n* ∈ {1, 2, …, 256} in the first GASSOM. Hence, the input vector to the second GASSOM is ${\;\text{x}}_{2,s,i}\left(t\right)\in {\mathbb{R}}^{1024}$ for a given scale *s* and patch *i*. The second GASSOM also contains *M* = 256 nodes, each with an associated 2-dimensional subspace spanned by the columns of the matrix ${\text{B}}_{2,s,m}\in {\mathbb{R}}^{1024\times 2}$ where *m* ∈ {1, 2, …, 256} indexes the node. As in Equation (2), the projections are computed by;

(4) $$\begin{array}{l} {\;\text{p}}_{2,s,m,i}\left(t\right)={\text{B}}_{2,s,m}^{T}{\;\text{x}}_{2,s,i}\left(t\right). \end{array}$$

Both the transition matrix and the node subspaces are learned online as the iCub behaves in the environment.

From the projections ${\;\text{p}}_{2,s,m,i}\left(t\right)\in {\mathbb{R}}^{2}$ at each node, we compute a feature vector $\phi_{s}\left(t\right)\in {\mathbb{R}}^{256}$ for each scale of the foveal image by computing the average squared length of the projections over all the patches.

(5) $$\begin{array}{l} \phi_{s}\left(t\right)=\left[\begin{array}{c} \frac{1}{P}{\sum_{i=1}^{P}\left\|p_{2,s,1,i}(t)\right\|}^{2}\\ \frac{1}{P}{\sum_{i=1}^{P}\left\|p_{2,s,2,i}(t)\right\|}^{2}\\ \begin{array}{c} \ldots \\ \frac{1}{P}{\sum_{i=1}^{P}\left\|p_{2,s,256,i}(t)\right\|}^{2} \end{array} \end{array}\right]. \end{array}$$

The final feature representation of the visual stimuli is the concatenation of the feature vectors at the two scales.

(6) $$\begin{array}{l} \phi_{\text{v}}\left(t\right)=\left[\begin{array}{c} \phi_{\text{fine}}\left(t\right)\\ \phi_{\text{coarse}}\left(t\right) \end{array}\right]. \end{array}$$

The proprioceptive feature vector $\phi_{\text{p}}\left(t\right)\in {\mathbb{R}}^{16}$ concatenates the arm position, velocity and acceleration measurements from the encoders, $\theta_{\text{a}}\left(t\right),{\dot{\theta }}_{\text{a}}\left(t\right),{\ddot{\theta }}_{\text{a}}\left(t\right)\in {\mathbb{R}}^{4}$ and the eye position and velocity${\;\theta }_{\text{e}}\left(t\right),{\dot{\theta }}_{\text{e}}\left(t\right)\in {\mathbb{R}}^{2}$. Each proprioceptive input is normalized by subtracting the mean and dividing by the standard deviation computed over the training data set.

### Eye motor controller

The eye controller maps the visual $\phi_{\text{v}}\left(t\right)\;\in {\mathbb{R}}^{512}$ and proprioceptive $\phi_{\text{p}}\left(t\right)\in {\mathbb{R}}^{16}\;$feature vectors to an eye acceleration command ${\ddot{\hat{\theta }}}_{\text{e}}\left(t\right)$ using the artificial neural network shown in Figure 1. Only one network is shown, corresponding to the generation of the acceleration command for one axis (pan or tilt). The other axis is controlled by a network with the same structure, but different weights.

Each neural network has 11 output neurons, corresponding to 11 possible acceleration actions, *a*_*i*_ ∈ **A** for *i* ∈ {0, 1, …, 11}, where;

(7) $$\begin{array}{l} \text{A}=\left\{-1.6,-0.8,-0.4,-0.2,-0.1,0,0.1,0.2,0.4,0.8,1.6\right\}\;deg/sample^{2}. \end{array}$$

The outputs of each neural network, π_*k,i*_(*t*) where *k* ∈ {pan, tilt} and *i* ∈ {0, 1, …, 11} encode the probabilities that the actions are chosen at each time *t*. Mathematically;

(8) $$\begin{array}{l} P\left[{\ddot{\hat{\theta }}}_{\text{e},k}\left(t\right)=a_{i}\right]=\pi_{k,i}\left(t\right), \end{array}$$

where ${\ddot{\hat{\theta }}}_{\text{e}}\left(t\right)={\left[\begin{array}{c} {\ddot{\hat{\theta }}}_{\text{e},\text{pan}}\left(t\right)\;{\ddot{\hat{\theta }}}_{\text{e},\text{tilt}}\left(t\right) \end{array}\right]}^{T}$. The eye acceleration command is generated by sampling from this probability distribution.

Within the neural network, the visual input first passes through a single fully connected 50 neuron hidden layer and the proprioceptive input first passes through two fully connected 25 neuron hidden layers with tanh activations before the two pathways are combined at the output layer, which is fully connected with a softmax output non-linearity. Mathematically;

(9) $$\begin{array}{l} \pi_{k,i}(t)=\frac{\exp(z_{k,i}(t)/\tau )}{\sum_{j=1}^{11}\exp(z_{k,j}(t)/\tau )}, \end{array}$$

where τ = 1 is a temperature parameter. The vector, ${\text{z}}_{k}\left(t\right)\in {\mathbb{R}}^{11}$, is given by;

(10) $$\begin{array}{l} {\text{z}}_{k}\left(t\right)={\text{W}}_{k,2}^{T}\tanh\left({\text{W}}_{k,1}^{T}\phi_{\text{v}}\left(t\right)\right)+{\text{W}}_{k,5}^{T}\tanh\left({\text{W}}_{k,4}^{T}\tanh\left({\text{W}}_{k,3}^{T}\phi_{\text{p}}\left(t\right)\right)\right), \end{array}$$

where ${\text{W}}_{k,1}\in {\mathbb{R}}^{512\times 50},{\text{W}}_{k,2}\in {\mathbb{R}}^{50\times 11},{\text{W}}_{k,3}\in {\mathbb{R}}^{16\times 25},{\text{W}}_{k,4}\in {\mathbb{R}}^{25\times 25}$ and ${\text{W}}_{k,5}\in {\mathbb{R}}^{25\times 11}$ are weight matrices. Our implementation includes constant bias terms at all layers, which we have not shown explicitly in the notation to avoid clutter.

The weights of the eye motor controller are learned online as the iCub behaves in the environment, using the natural actor-critic reinforcement learning algorithm (Bhatnagar et al., 2009). The network generating the probabilities described above is the actor (policy) network. The actor-critic algorithm also requires a second network to approximate the value function, which depends upon both **ϕ**_v_(*t*) and **ϕ**_p_(*t*). We use a single layer linear network with 528 inputs feeding into a single linear output neuron for the critic network.

The instantaneous reward is given by;

(11) $$\begin{array}{l} r\left(t\right)=-\frac{1}{2}\left(e_{\text{fine}}\left(t\right)+e_{\text{coarse}}\left(t\right)\right), \end{array}$$

where;

(12) $$\begin{array}{l} e_{s}\left(t\right)=\frac{1}{P}\sum_{i=1}^{P}\underset{n}{\text{max}}{\left\|p_{1,s,n,i}(t)-\;p_{1,s,n,i}(t-1)\right\|}^{2}, \end{array}$$

for *s* ∈ {fine, coarse}. This reward penalizes changes in the patch projections, which should be constant if the image of the end effector is stabilized.

### Training and testing environment

**Figure 3:**
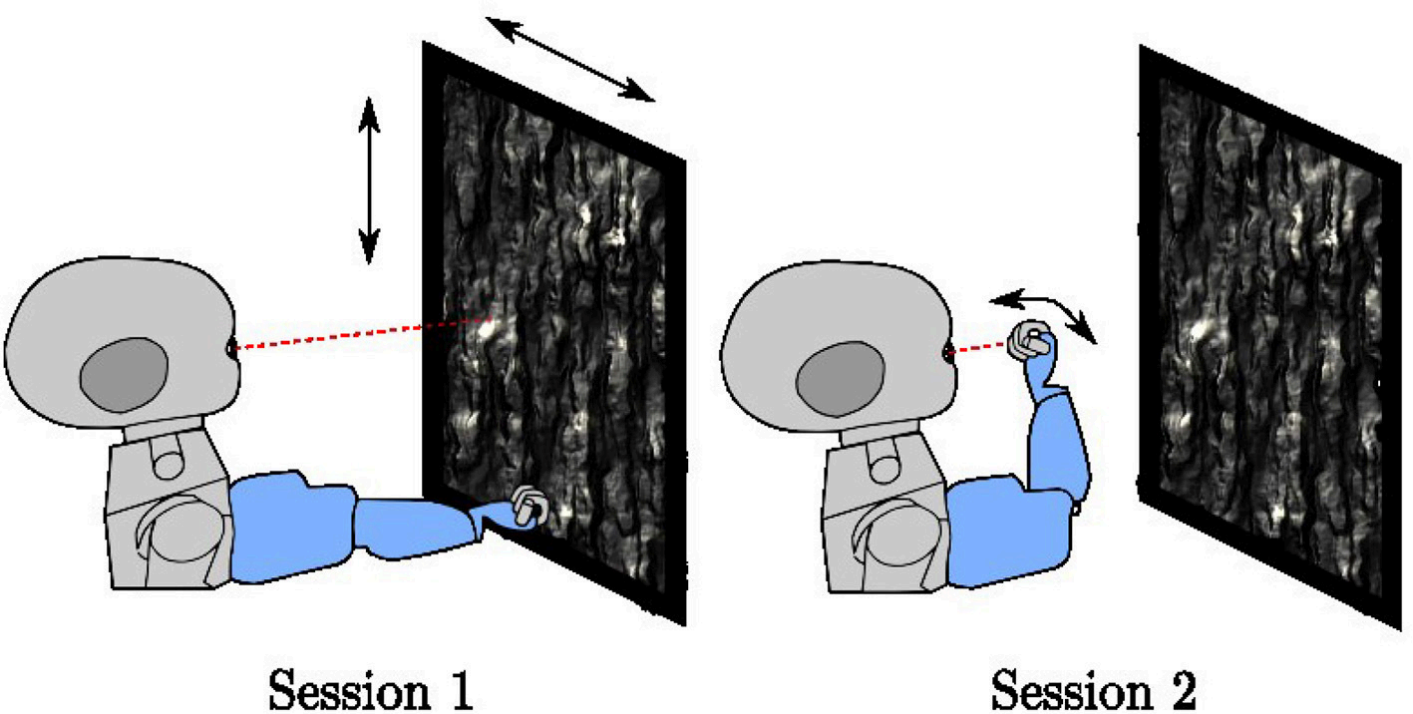
The two-session types used in training, In session type 1, the arm rests in a fixed position outside the field of view of the robot while a textured plane moves in the background. In session type 2, the arm babbles in front of the robot with the textured plane remains stationary.

Training runs for a total of 400,000 iterations. It interleaves two different types of sessions (Figure 3), each lasting 5,000 iterations at a time. For both session types, the environment contains a planar object mapped with a natural image texture chosen at random from the (Olmos and Kingdom, 2004) database of size 2142 × 1422 pixels located at 0.4–0.8 m distance in front of the iCub. When placed directly in front of the robot, the plane subtends 136° of visual angle horizontally and 118° of visual angle vertically. The texture is changed every 500 iterations. In session type 1, the arm remains stationary in a position where it is outside the field of view of the robot. Only the textured plane, which moves randomly horizontally and vertically, is visible. In session type 2, the robot arm babbles so that the end effector moves randomly in front of the iCub. Depending on the eye and arm position, the center of the eye gaze may fall on the robot arm or on the resting plane. If the eye gaze falls on the center of the palm of the iCub, the hand fills both the coarse and fine scale foveal windows, but this is not always the case. This training setup is intended to mimic a general environment, where an agent is exposed to both self-generated and exteroceptive motion in the visual environment.

In session type 2, we use motor babbling to generate the visual and proprioceptive data for the learning algorithm. The arm babbles around a home pose $\theta_{\text{a}}^{\text{h}}=\left[-82^{o}\;22^{o}\;40^{o}\;90^{o}\right]$, which is chosen so that the center of the iCub's hand falls on the image center of the right eye when its pan and tilt angles are zero. The arm moves through a randomly generated trajectory (${\hat{\theta }}_{\text{a}}\left(t\right)$ in Figure 1) in the arm joint space. The babbling trajectory is generated by feeding a set of via points sampled from a uniform distribution $\left[\theta_{\text{a},\text{i}}^{\text{h}}-12^{o},\theta_{\text{a},\text{i}}^{\text{h}}+12^{o}\right]$ for *i* ∈ {1, 2, 3, 4} into the “mstraj” function of the Robotics Toolbox (Corke, 2017) to generate a trajectory consisting of linear segments connected by polynomial blends.

In session type 1, the planar object follows a trajectory created by first generating an arm trajectory as described above, and then moving the plane so that its center point follows the same angular trajectory as the center of the iCub's hand. This ensures that the statistics of the visual motion induced by the plane are similar to the statistics of those induced by the hand.

The eye rotational angles are restricted to ±40^*o*^ and ±30^*o*^ in pan and tilt, respectively. The rotational velocity of the eye is also limited to ±3 deg/sample in both pan and tilt. During training, we reset the eye position to a “home” position $\theta_{\text{e}}^{\text{h}}=\left[0^{o}\;0^{o}\right]\;$ and the velocity to zero every 500 iterations. This ensures the eye orientation does not drift off so far that the eye never sees the hand. For each trajectory during testing, we initialize the eye velocity to zero and the eye position so that the gaze vector intersects the center of the palm.

We chose this method of random babbling and trajectory generation for its simplicity. There are a number of ways we can make the motion more biologically realistic, e.g., through the use of dynamic movement primitives (Schaal, 2006) for trajectory generation, or through the use of goal babbling (von Hofsten, 2004) to choose the via points. The use of dynamic movement primitives would alter the statistics of the image motion induced by the hand, which might change the smooth pursuit performance. The use of goal babbling might improve the speed of learning (Baranes and Oudeyer, 2013). These would be interesting extensions of the model to investigate. However, we do not expect their incorporation to change the main qualitative findings we report here.

For the sake of simplicity in our simulations, we use the iCub robot simulator to take into account the kinematics of the iCub robot as well as to model the geometry and appearance of the visual environment. We do not take into account the dynamics of the robot, nor do we incorporate a biologically realistic model for the eye movement dynamics. Rather, in each iteration, we move the robot arm to the configuration determined by the random babbling arm motion via position control. We assume that the eye velocity command is executed perfectly, by determining the location of the eye in the next iteration as the sum of the encoder measurement of the current position plus the velocity command in Equation (1), and move the eye there via position control. The images taken by the iCub in the new arm/eye positions determine the next visual input to the model. The proprioceptive input is determined from the motor encoders and their first and second differences in time. We believe that incorporating more realistic models of arm and eye dynamics are a natural next step. If these models are more biologically realistic, the model may give a better quantitative account of the performance of human subjects.

## Results

### Learned visual representation

**Figure 4:**
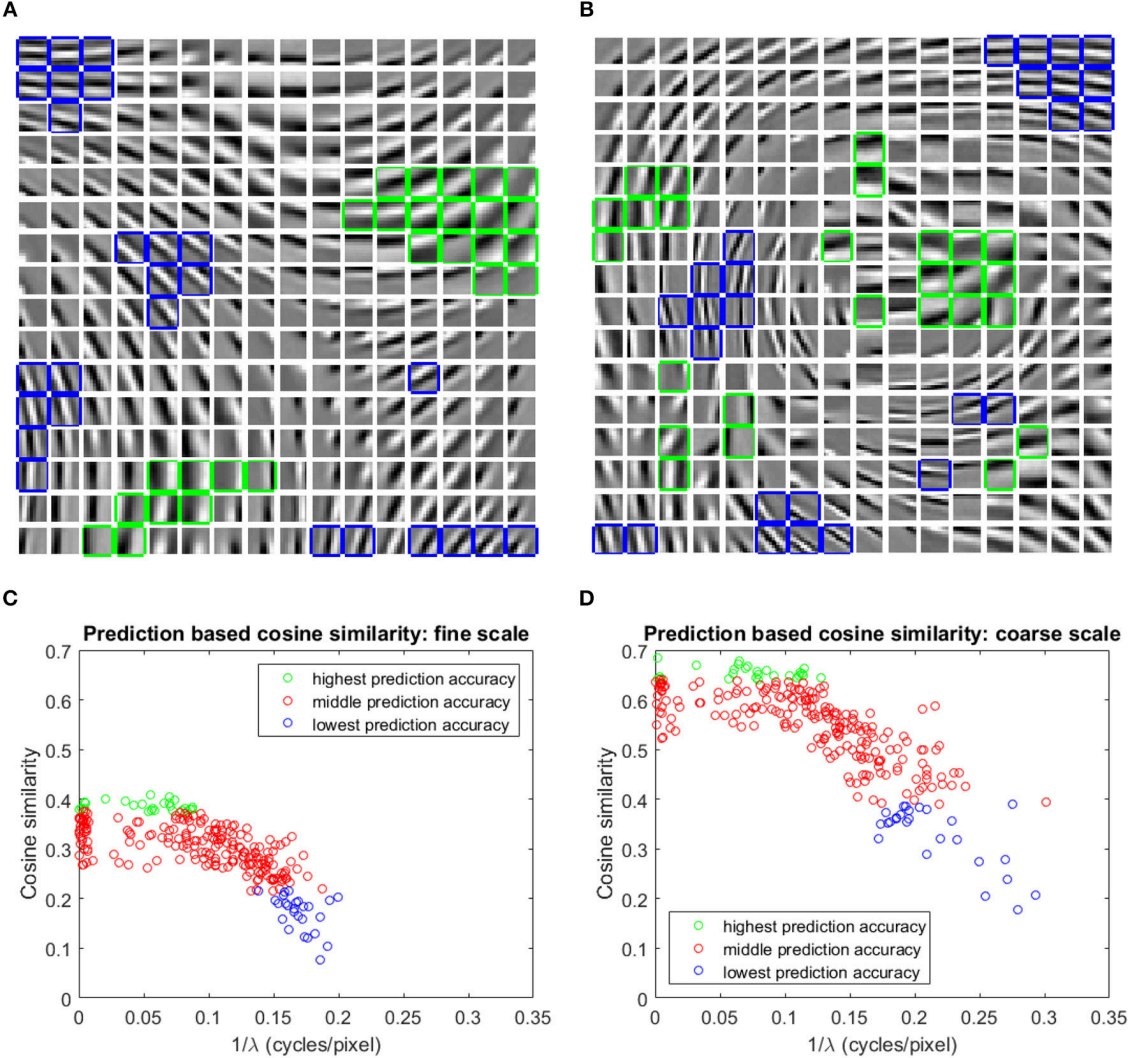
**(A,B)** One out of each pair of basis vectors spanning the 256 subspaces in the fine **(A)**, and coarse **(B)** scale GASSOMs. The basis vectors are shown as a **16 × 16** array of **10 × 10** images. In each subplot, the twenty six basis vectors of the subspaces with the highest (lowest) average cosine similarity between the predicted and actual outputs are outlined in green (blue). **(C,D)** Scatter plots of the average cosine similarity of the prediction vs. best-fit spatial frequency for the fine **(C)**, and coarse **(D)** scale basis vectors.

The basis vectors in the first stage GASSOM are analogous to the receptive fields of orientation-tuned simple cells in the human primary visual cortex (Chandrapala and Shi, 2015). As shown in Figures 4A,B, the basis vectors of the first GASSOM are tuned to specific orientations and spatial frequencies. The basis vectors corresponding to fine and coarse scales have similar characteristics. The corresponding basis vectors associated with a node in the first GASSOM have a phase difference close to 90^*o*^ to represent the two orthogonal basis vectors. We initialize these basis vectors with the basis vectors learned on natural images. In addition, the parameters corresponding to the first GASSOM are fixed during the visual representation learning.

The prediction module predicts the projections at time *t* of the first stage GASSOM given the projections at time *t*−1 and the proprioceptive inputs. We evaluate the accuracy of the prediction module using the average cosine similarity between ${\;\hat{\text{p}}}_{1,s,n,i}\left(t\right)$ and **p**_1,*s,n,i*_(*t*). For this test, the eye and the arm are moved independently of each other. The eye velocities are sampled from Gaussian distributions fitted to the distribution of the eye velocity during training in both pan and tilt directions. The standard deviations of the fits are $\sigma_{{\dot{\theta }}_{\text{e},\text{pan}}}=0.7337$ deg/sample (*r*^2^ = 0.982) and${\;\sigma }_{{\dot{\theta }}_{\text{e},\text{tilt}}}=0.6387$ deg/sample (*r*^2^ = 0.989). Arm trajectories are generated in a similar way to the training. In order to maintain the gaze on the robot hand, we execute a saccade to bring the eye gaze back to the center of the hand once the eye gaze drifts outside of the arm region. We evaluate the predictors over 10,000 iterations. The basis vectors from the subspaces with the highest and lowest average cosine similarity for two scales are outlined in green and blue in Figures 4A,B.

We fit a two-dimensional Gabor function to each basis vector to identify the factors influencing the prediction accuracy. Figures 4C,D show that the average cosine similarity of a predictor is related to the spatial frequency ($\frac{1}{\lambda }$; where λ is the spatial wavelength) of the basis vector. Higher spatial frequencies have a lower cosine similarity for predictors in both fine and coarse scales. Intuitively, basis vectors with higher spatial frequencies are more sensitive to retinal motion than those with low spatial frequencies. The transformations associated with higher spatial frequency basis vectors are more difficult to predict. The cluster of data points close to $\frac{1}{\lambda }=0$ in Figures 4C,D are basis vectors whose fitted Gabor functions had very long spatial wavelengths. These typically corresponded to basis vectors with main support near the edges of the patch.

The second stage GASSOMs jointly encode the actual and predicted projections onto the subspaces of the first GASSOM. If the predictions from knowledge of the proprioception are accurate, there should be little difference between the actual and predicted projections. Differences between the two arise due to exteroceptive motion, which cannot be predicted from proprioception, as well as inaccuracy in the predictor. The basis vectors in the second GASSOM encode these differences and inherit orientation and spatial frequency tuning from the first stage. Encoding only the residual motion after prediction helps to reduce correlation between the visual and proprioceptive cues.

We examine the tuning of the basis vectors in the second GASSOM using drifting two-dimensional cosine gratings in 10 × 10 pixel image patches. For this test, we fix the proprioceptive input to zero self-motion. We record the responses from the subspaces of the second GASSOM to all combinations of motion, spatial frequency and orientation, where spatial wavelengths varied from 3 to 20 pixels, motion from −2 to 2 deg/sample, orientation from 0° to 180°. For each subspace, we determine the preferred tuning from the combination that resulted in the maximum magnitude response.

**Figure 5:**
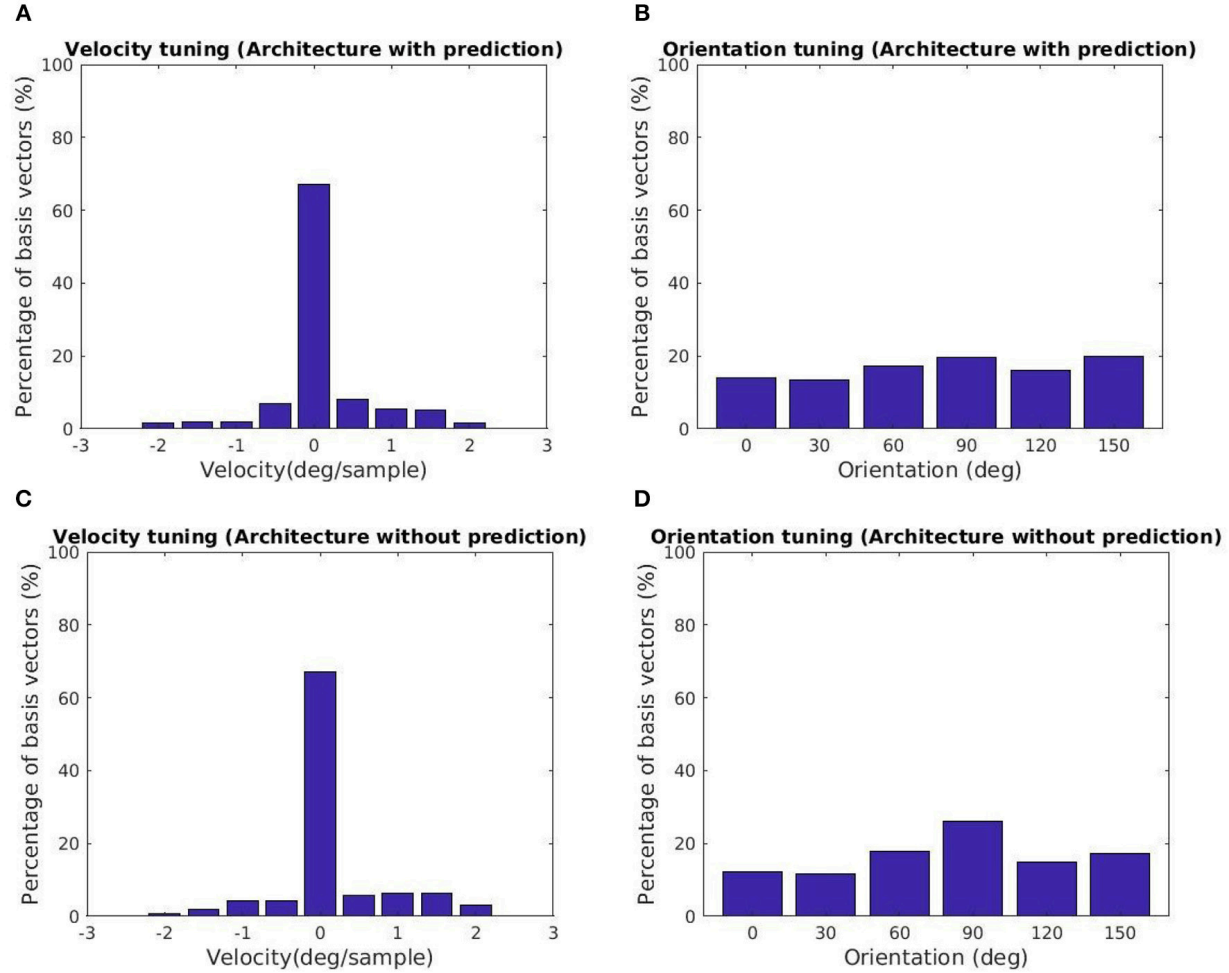
Tuning statistics for the second stage GASSOM. **(A,B)** (Velocity, Orientation) statistics for the architecture with the prediction module. **(C,D)** (Velocity, Orientation) statistics for the architecture without prediction. The majority of the basis vectors in the second GASSOM are tuned in to zero retinal slip in both cases.

The tuning characteristics for the fine scale basis vectors are provided in Figures 5A,B. Here, we also present the tuning statistics corresponding to a model without the prediction module shown in Figures 5C,D. The majority of the basis vectors are tuned to zero velocity for both architectures as shown in Figures 5A,C. The tuning velocities of the architecture with, without prediction have a variance of 0.3660, 0.4286 (*deg*/*sample*)^2^. The tuning orientations are distributed close to a uniform distribution as shown in Figures 5B,D. The KL divergence with the uniform distribution of orientations for the architecture with, without prediction is 0.0113, 0.0397. Hence, the two architectures prefer zero retinal slip for all the tuning orientations. For the architecture with prediction, the variance of the tuning velocities is lower compared to the other architecture.

### Learning the eye motor controller

The eye controller is tested by evaluating the tracking performance for a set of 10 different arm trajectories, each lasting 1,000 iterations. The trajectories are generated in the same way as in training. The performance is measured by computing the root mean squared error (RMSE) between the target and actual eye velocities.

**Figure 6:**
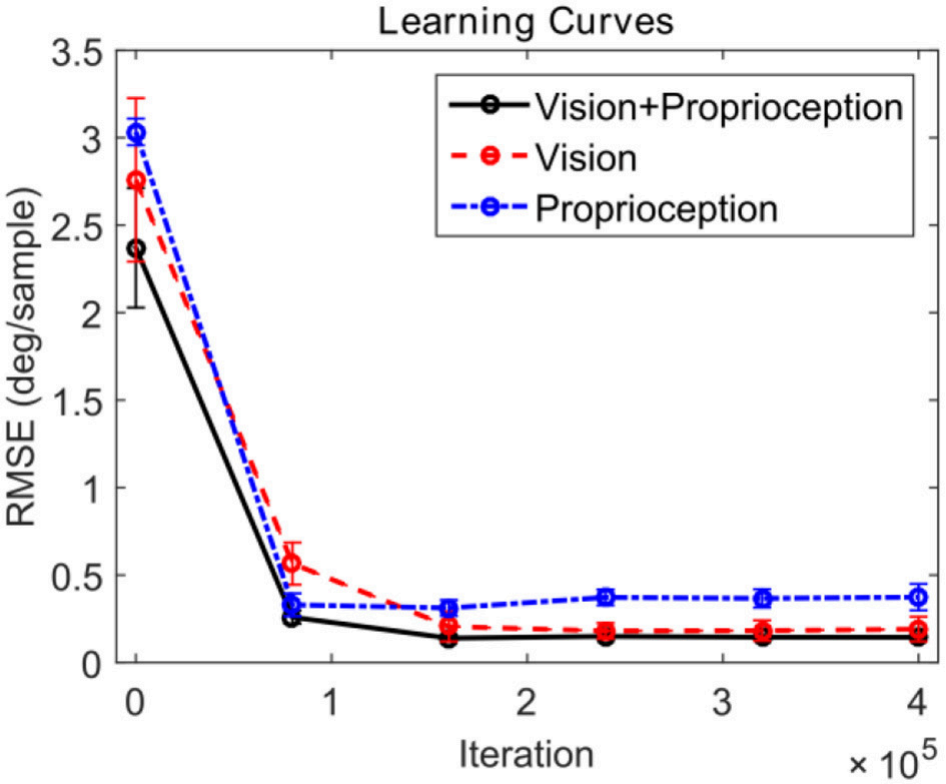
Learning curves corresponding to three testing scenarios. The performance for pan and tilt angular velocities are averaged. The black solid line shows the learning curve corresponding to the system using both visual and proprioceptive inputs to track the robot hand. The red dashed line shows the learning curve corresponding to the system using the pure visual input to track the robot hand. The blue dash-dot line shows the learning curve corresponding to the system using pure proprioceptive input.

We compare the performance of the system in three different scenarios. In all cases, the robot attempts to track the end effector of the robot. In the first case, the model is driven by both visual and proprioceptive stimuli. In the second case, the model is purely driven by vision, with illusory proprioceptive input being provided that suggests that the arm is fixed at the resting position outside the field of view used in training session type 1. In the third case, the model is purely driven by proprioception, with the visual feature **ϕ**_v_(*t*) replaced by the expected value computed over time *E*[**ϕ**_v_(*t*) ]. This approach allows us to compare the three cases by providing the same visual stimuli (the robot hand) in distinct scenarios. Figure 6 depicts the learning progress recorded at 6 checkpoints occurring every 80,000 iterations. The RMSE for both pan and tilt angular velocities are averaged over 30 different trials comprising 10 trajectories and 3 different training trials. The learning curves in Figure 6 illustrate that in steady state, using both visual and proprioceptive stimuli is much more accurate than using either stimuli alone. This is typical of multimodal integration.

### Comparison with psychophysical experiments

**Figure 7:**
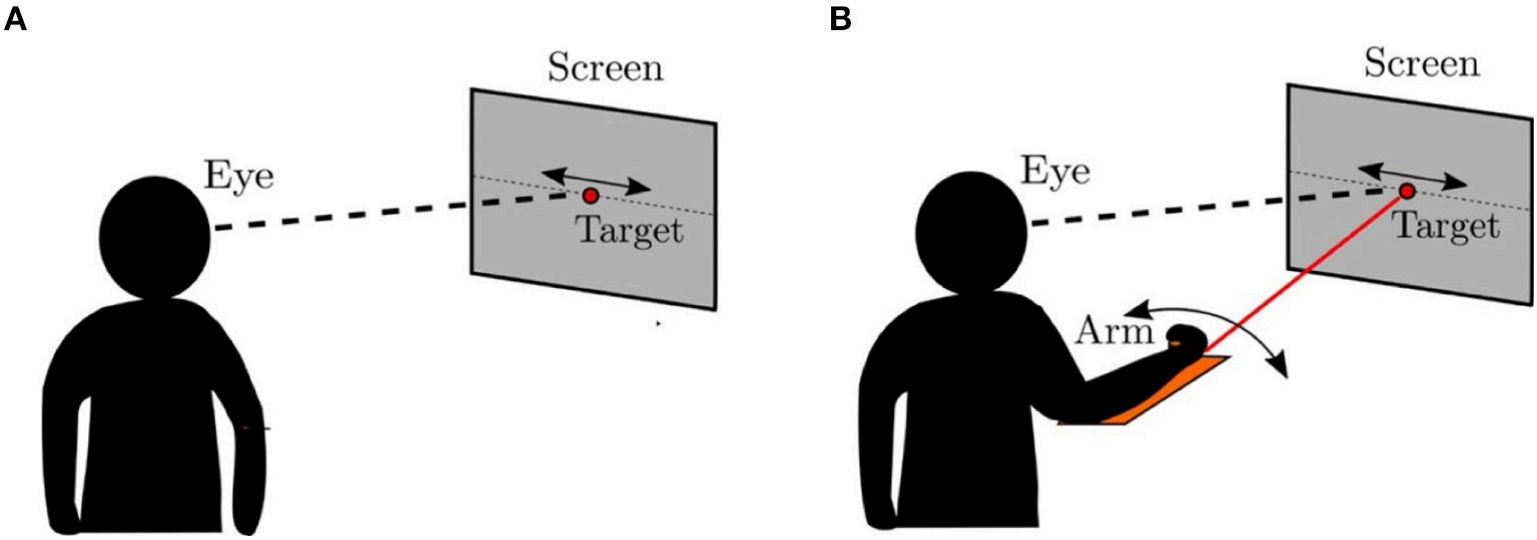
Psychophysical experiment (Vercher et al., 1993). **(A)** The target was controlled externally. The subject was asked to track the target using direct visual input. **(B)** The target was controlled by the subject using a lever. The subjects simultaneously tracked the target ulitlizing both visual and proprioceptive inputs.

In this section, we compare the eye movements generated by the model to that of the human oculomotor system using an experimental protocol similar to that described in Vercher et al. (1993), which is illustrated in Figure 7. Vercher et al. measured the frequency responses of the human oculomotor plant during visual tracking for five subjects (4 males and 1 female) in two different cases. In the first case, the subject was tracking a target moving in a sinusoidal trajectory as shown in Figure 7A. In the second case, the target trajectory was controlled by the subject using his/her arm while tracking the corresponding target with the eyes as shown in Figure 7B. The eye movements in these two cases were compared to understand the role of proprioception in oculomotor control.

**Figure 8:**
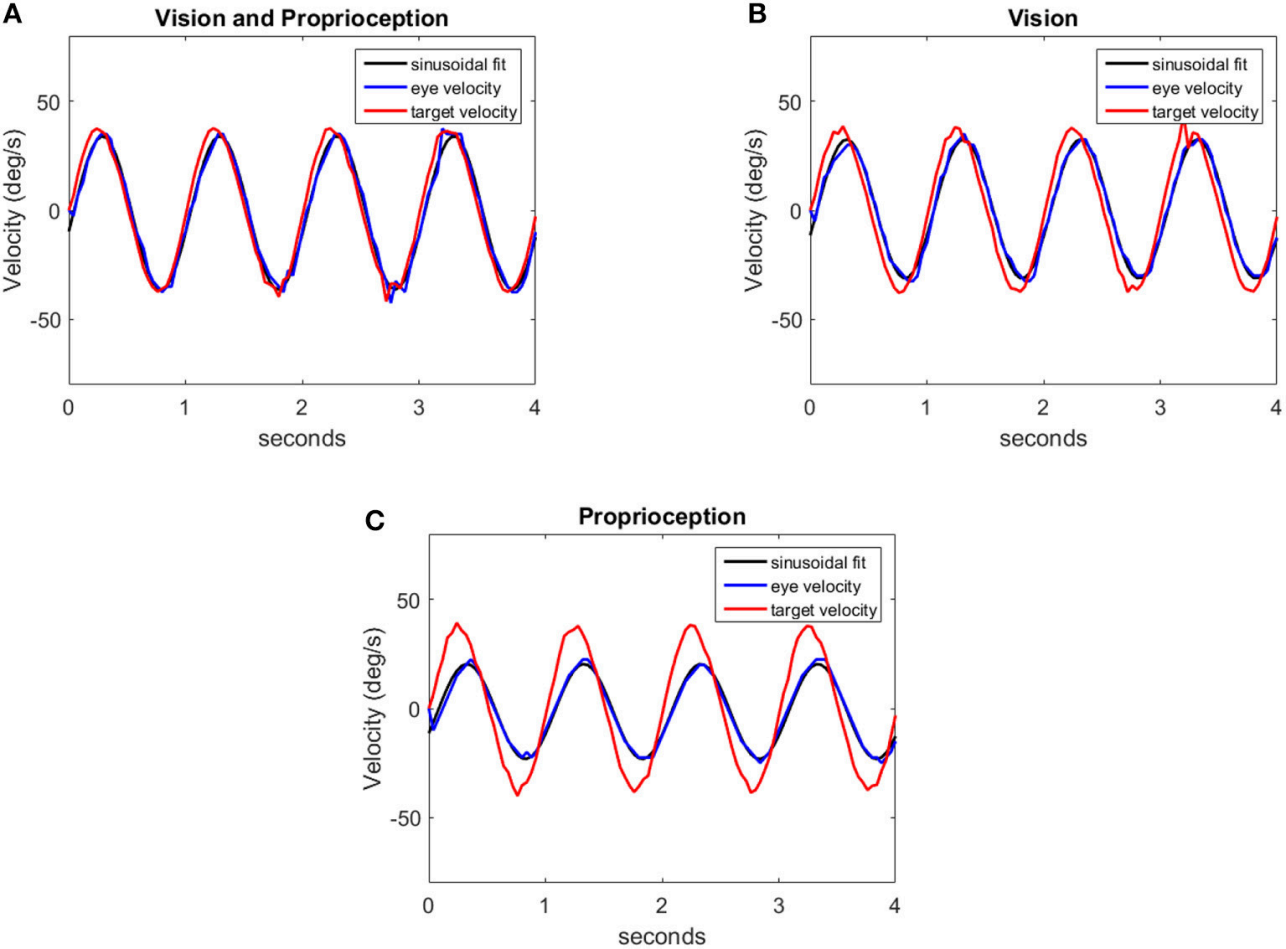
Eye velocity trajectories corresponding to the eye responses for different sensory modalities: **(A)** Vision and Proprioception, **(B)** Vision, **(C)** Proprioception. Each figure contains a trajectory (red) indicating the target velocity, the respective eye velocity (blue) and the sinusoidal fit (black) for the eye velocity.

In our experiment, with the model to generate comparable data, we use the end effector of the robot arm as the target. The end effector is moved in a sinusoidal trajectory between −6^*o*^ and +6^*o*^ in the pan direction with respect to the eye. The frequency of the motion varies from 0.5 to 2 Hz in 0.5 Hz steps. Sample trajectories generated by the model at 1 Hz frequency are shown in Figure 8. We fit a sine function to the eye velocity to compute the velocity gain and phase difference with reference to the target trajectory. The eye trajectory in Figure 8A shows a higher gain and a lower phase delay compared to the trajectory in Figure 8B. Hence, the addition of proprioceptive information improves the velocity gain in comparison to vision alone. The addition of proprioception also reduces the phase delay. Our system is also able to move the eye solely with the proprioceptive input as illustrated in Figure 8C.

**Figure 9:**
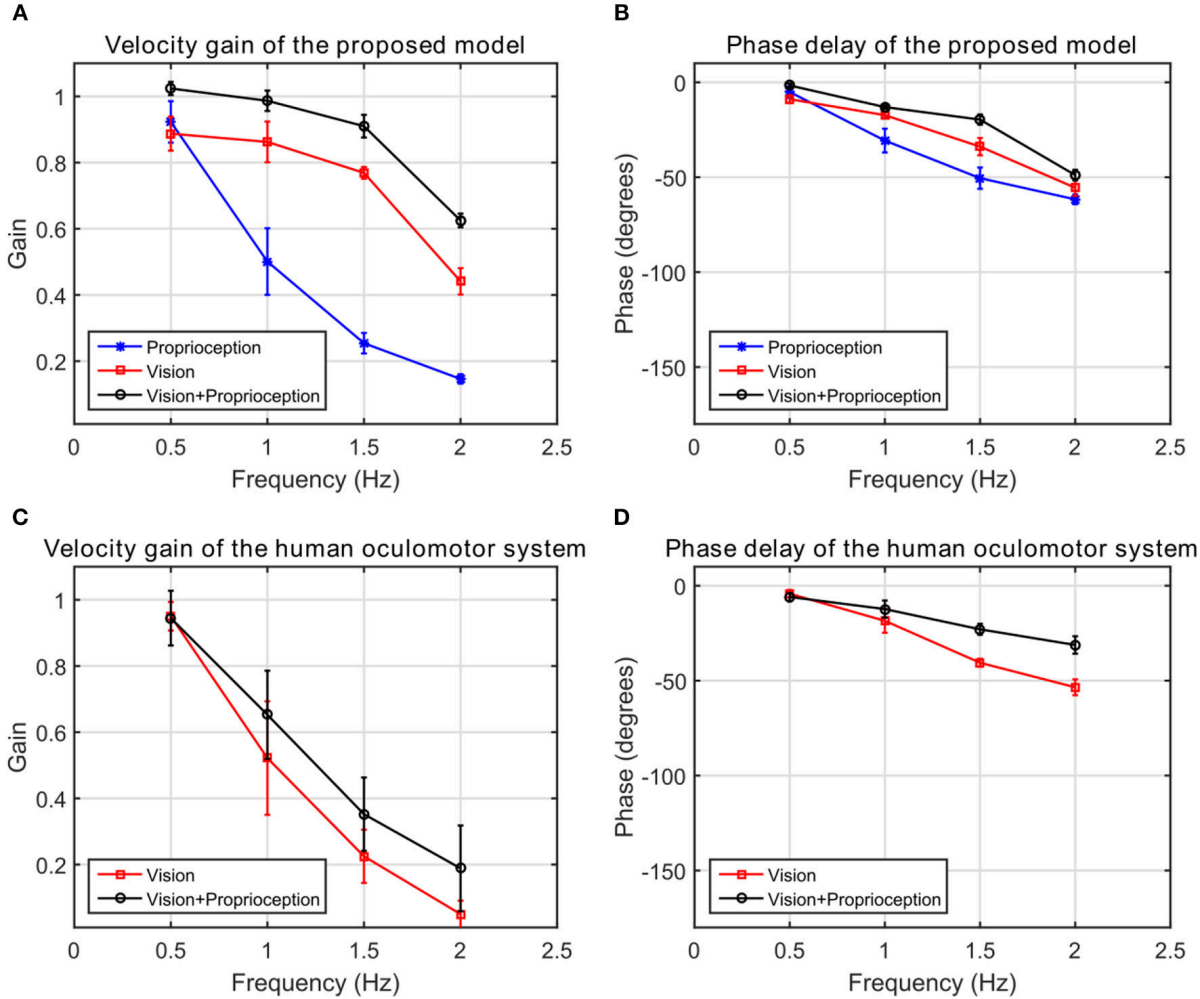
The frequency response of the human oculomotor system compared with the frequency response of the proposed model. **(A)** The velocity gain of the proposed system. **(B)** The phase delay of the proposed system. **(C)** The velocity gain of the human oculomotor system. **(D)** The phase delay of the human oculomotor system.

We compare the results of our model with human performance, by extracting the frequency response data from Figures 2, 3 in Vercher et al. (1993), which show the eye velocity gains and phase delays averaged across the five subjects. Figure 9 compares the frequency responses. The model and human data have qualitative similarities. According to the gain plots in Figures 9A,C, both responses have a higher gain in the presence of both vision and proprioception. In addition, proprioception combined with vision has a lower phase delay as shown in Figures 9B,D. For the subjects in this study, both efferent and afferent information is available during tracking of the self-moved target, whereas our model only includes afferent information from proprioception. Vercher et al. (1996) studied the role of proprioception in eye-hand coordination by comparing the behavior between deafferented and control subjects. The deafferented subjects showed little difference in performance between tracking a self-moved target and an external target. This highlights the importance of proprioception as a non-visual signal for smooth pursuit control.

### Qualitative evaluation of performance

To observe the tracking performance qualitatively, we generate a video comparing the eye trajectories as the arm moves randomly for the three different combinations of cues^1^ This video demonstrates the performance by projecting the gaze position to an image frame obtained from a fixed camera for three different testing cases. The right eye camera is moving differently in all three testing cases. Relating the gaze to a reference camera frame makes it easier to compare the different cases. From the video, the pure proprioception based tracking underperforms in comparison to the other two cases. This is consistent with the velocity gain and phase delay responses in Figure 9.

**Figure 10:**
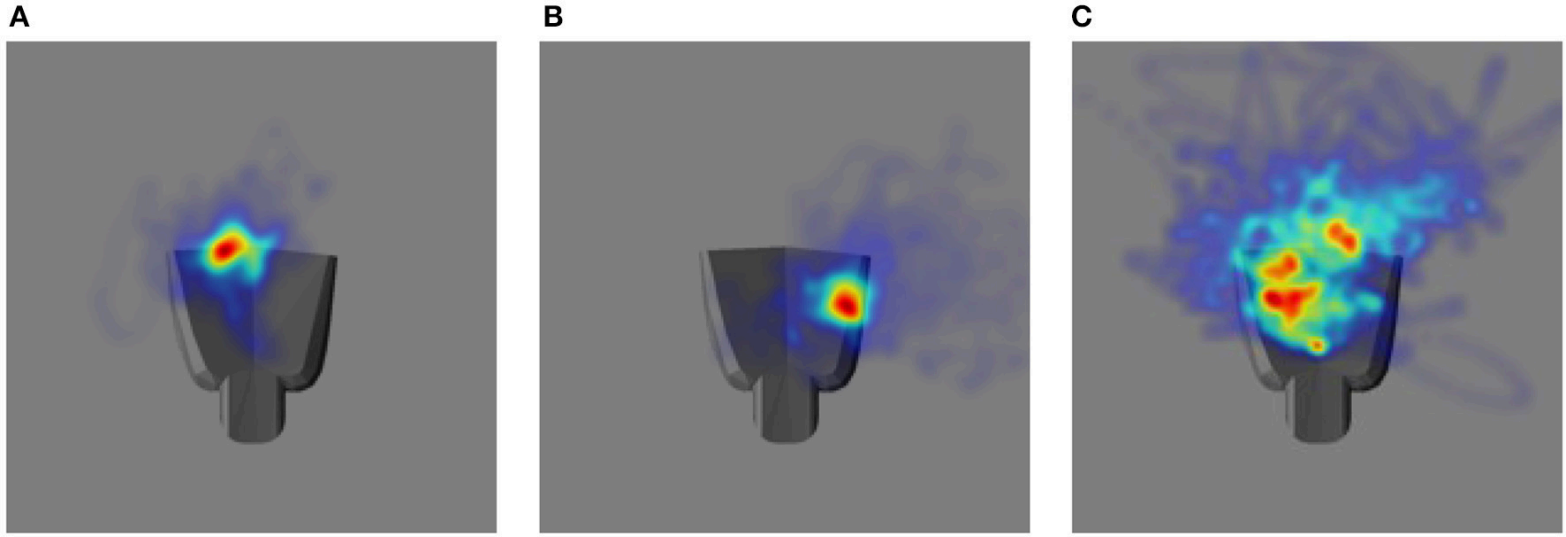
The heat map illustrating the distribution of eye gaze intersection with a plane parallel to the palm of the robot hand. The robot hand is superimposed to each image to have a qualitative comparison: **(A)** Vision and Proprioception **(B)** Vision **(C)** Proprioception.

The performance in the video can be summarized by projecting the gaze vectors to the end effector coordinate system. The origin of this coordinate system is located on the palm of the robot end effector. Figure 10 shows distributions of the intersections between the eye gaze direction and the plane passing through the origin parallel to the palm surface over the entire tracking trajectory as two-dimensional heat maps, one for each of the testing cases. In comparison to Figures 10A,B, 10C shows larger variability in the gaze position on the end effector. This is consistent with the poorer tracking performance for proprioception alone than in the other two cases. The model does not explicitly define a precise end effector location to be tracked. Since only acceleration commands are generated, the gaze position can drift. Thus, the mean gaze position projected to the palm varies across different training trials for the three different testing cases.

We also illustrate the system's robustness to changes in the visual appearance of the robot end effector qualitatively through an accompanying video^2^ We change the appearance by moving the wrist and fingers of the iCub with sinusoidal joint trajectories. No information about finger and wrist motion is provided to the system. When eye acceleration is driven by vision alone, the eye drifts away faster from the end effector. The changes in the appearance of the end effector introduce visual perturbations, which are challenging to follow in comparison to changes due to translation of the end effector. For the other two cases, the proprioceptive inputs, which are not altered due to the changes in the visual appearance of the robot end effector, enable the robot to maintain the gaze on the palm for a longer time.

### Importance of the prediction module

**Figure 11:**
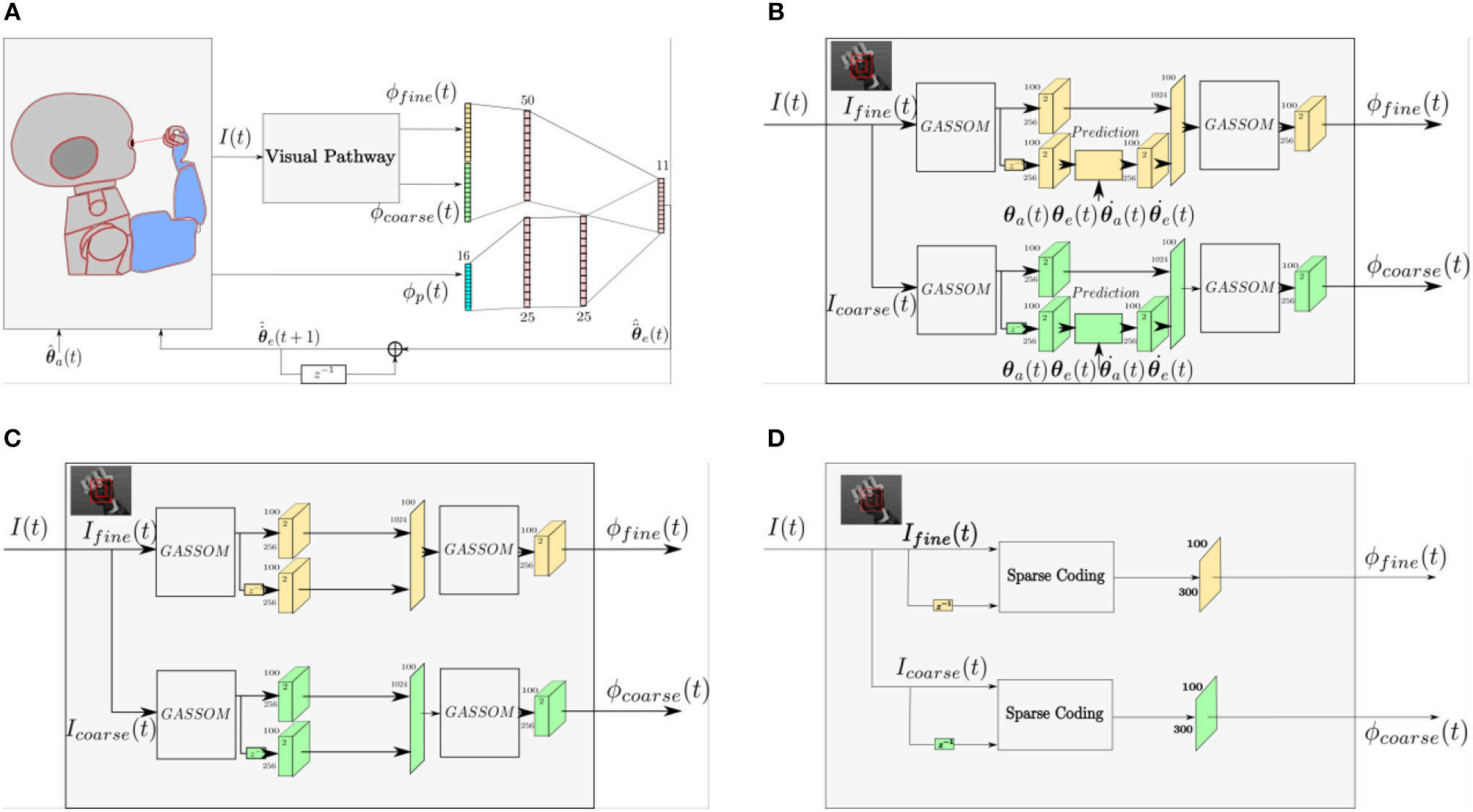
**(A)** A general architecture representing all of the architectures compared in this study. **(B)** The prediction based visual pathway proposed in this paper. **(C)** The visual pathway without the prediction module. **(D)** A Sparse Coding based visual pathway.

To identify the role of the prediction module in the proposed model, we compare the performance of three different models. Figure 11A shows a general architecture that covers the three models compared in this study. The three models differ only in their visual pathways. The proprioceptive features and the eye controller neural network are identical. Figure 11B illustrates the prediction based visual pathway proposed in this paper. The pathway in Figure 11C has a very similar structure, except that the prediction module is removed. Comparing these two models enables us to identify the benefit of the prediction module. The pathway shown in Figure 11D is the sparse coding based visual representation used in our earlier work (Wijesinghe et al., 2017).

**Figure 12:**
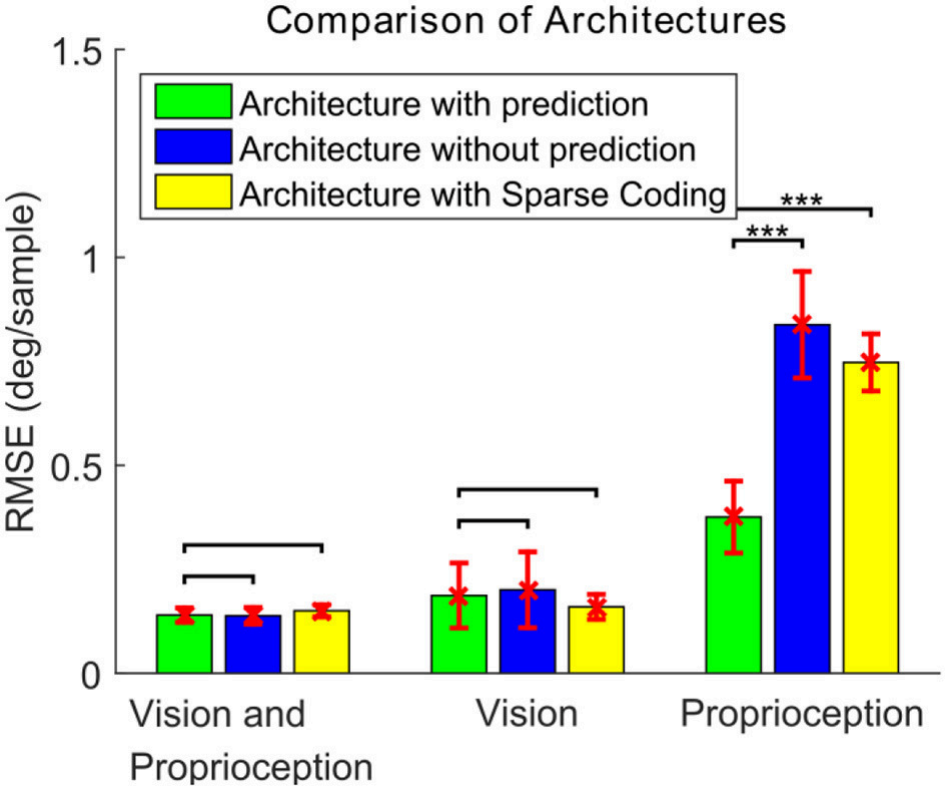
The RMSE (mean ± standard deviation) of the eye velocity compared to a target velocity for three different architectures and three different combinations of sensory modalities. For the paired *t*-test comparisons, no mark indicates differences are not statistically significant (*p* > 0.05), ***indicates statistical significance (*p* < 0.001).

To compare the performance, each model is tested for 20 different testing trials (10 different trajectories for each of the two training scenario types). Figure 12 shows the RMSEs between the target and actual eye velocities. We perform paired sample *t*-tests to compare the RMSE of the model with prediction with the RMSEs of the other two. The effect size is also computed according to the Cohen's d formula. The Differences between the performance of the models in Figures 11B,C are not statistically significant for vision and proprioception (*p* = 6.27 × 10^−1^, effect size = −0.0876) nor vision alone (*p* = 4.40 × 10^−1^, effect size = 0.1619). The model with prediction performs significantly better for proprioception alone (*p* = 6.16 × 10^−10^, effect size = 4.2334). We have similar findings for the Sparse Coding based model in Figure 11D. Differences in performance are not significant for vision and proprioception (*p* = 7.29 × 10^−2^, effect size = 0.6531) nor vision alone (*p* = 1.49 × 10^−1^, effect size = −0.4611). The model with prediction performs significantly better with proprioception alone (*p* = 5.94 × 10^−15^, effect size = 4.7588).

These results demonstrate that the proposed prediction based model exhibits superior performance compared to models without prediction. We attribute this to reduced correlation between the visual and proprioceptive features in the prediction-based model.

## Discussion

In this article, we propose a model based on the Active Efficient Coding (AEC) framework that enables a robot to learn to track its end effector using a combination of visual and proprioceptive cues. Rather than simply concatenating the two sets of features, the proposed model predicts visual consequences of actions, which removes information correlated with proprioception from vision. The model enables a robot to learn to track an object for three cases: using both visual and proprioceptive cues corresponding to the typical case of end effector tracking, using only visual cues corresponding to tracking of an external independently moving object, and using pure proprioception corresponding to tracking of the end effector in darkness.

The incorporation of prediction is motivated by recent studies on neural responses in V1 of mice during locomotion (Niell and Stryker, 2011; Keller et al., 2012; Attinger et al., 2017). These studies suggest that the responses of cells in the V1 depend upon predictions of the sensory consequences of motor actions. In fact, locomotion improves the encoding of visual stimuli (Dadarlat and Stryker, 2017). In our model, the visual representation encodes the residual motion after removing the predicted effects of self-motion from the observed visual flow. We show that the proposed prediction module has the ability to predict the visual sensory consequences of proprioceptive inputs (Figure 4). Specifically, the visual sensory consequences for subspaces with low spatial frequency basis vectors in the first stage GASSOM are easier to predict compared to higher spatial frequencies.

The incorporation of learning into both the perception and action components of the perception-action loop in this model allows the sensory representation and the action generation network to co-adapt as the agent behaves in the environment. We characterized the performance of the eye controller both quantitatively and qualitatively. Using both visual and proprioceptive sensory stimuli to drive eye motion results in more accurate tracking of the end effector compared to using either sensory stimulus alone. Moreover, the inclusion of proprioception also makes the model more robust to changes in the appearance of the end effector.

We compare predictions of our model with findings from human psychophysical experiments studying the contribution of proprioception to human oculomotor control. Our results in Figure 9B suggest that incorporating proprioception reduces phase delay. This characteristic of the human oculomotor system has been found repeatedly. First, early work analyzing self-moved targets showed that the information about arm motion plays an important role in self-motion tracking (Steinbach and Held, 1968). The eye motion lagged behind target motion less for active arm motions than passive arm motions. Second, active and passive hand motion tracking along with tracking of external visual targets were qualitatively compared in Mather and Lackner (1980). External target tracking used a larger number of saccades per cycle and had larger latency compared to the oculomotor tracking of the hand. The external target tracking was more challenging since the target motion was unpredictable. Third, experiments conducted by Gauthier et al. (1988) quantitatively compared eye tracking of an external object and of the subject's hand. The average latency when the eye tracked an external object (150 ± 30 ms) was much longer than the average latency when the eye tracked the hand (30 ± 10 ms). This delay was also prominent in the onset of smooth pursuit eye motions (Domann et al., 1989). Finally, Chen et al. (2016) showed that eye precedes a target controlled by the finger in congruent pursuit in comparison to opposite movements. However, our model does not exhibit this property.

Our results qualitatively agree with multiple psychophysical studies showing that proprioception can also change the velocity gain of the human oculomotor plant. During eye tracking of an external object, the eye velocity saturated at very low velocities around 40 deg/s compared to 100 deg/s during eye tracking of the hand (Domann et al., 1989). As shown in Figure 9A, velocity gains are larger at higher frequencies when the robot tracks its hand compared to an external object. Our model demonstrates that with the assistance of proprioception, the model is capable of maintaining a higher tracking gain for high frequency stimuli. This property is qualitatively consistent with the human oculomotor system (Vercher et al., 1993).

Human eye motion is mainly driven by visual stimuli. However, non-visual signals can also drive eye motion in certain tasks (e.g., in the darkness). Figure 8C illustrates that for our model proprioception alone has the capability to elicit eye movements. Several articles have studied the contribution of non-visual signals to oculomotor control. First, Steinbach (1969) showed that proprioceptive inputs alone were sufficient to generate smooth pursuit eye movements. Second, smooth pursuit movements generated by non-visual stimuli were studied in Berryhill et al. (2006). Tracking a pendulum in the darkness, proprioceptive stimuli had a velocity gain close to 0.3. On the other hand, with direct visibility of the pendulum, the subject had the ability to track the pendulum more accurately with a velocity gain of 0.7. Our model exhibits similar qualitative changes in visual vs. proprioceptive gain.

Although our model exhibits similar qualitative characteristics as human oculomotor tracking, it does not match quantitatively. In particular, the velocity gain of the model is much higher than that of the human oculomotor system (Figure 9). This mismatch may arise because we assume in our simulations that the eye velocity command generated by the model is executed perfectly by the eye. A more realistic model would include processing and propagation delays, a more realistic model of the neural control of eye velocity, as well as a dynamical model of the physical oculomotor plant. For example, the cerebellum is a part of the neural circuit for eye-hand coordination for oculomotor control (Miall et al., 2001), but this is not reflected in the proposed model. We anticipate that the incorporation of these elements into future extensions of the model would degrade the velocity gain observed during tracking, bringing the model simulations into closer quantitative agreement with human performance.

This work can be extended in several directions. First, the model could be extended to include saccadic eye movements. Tracking targets with the eyes typically consists of a combination of pursuit and saccadic eye movements. For example, the majority of the eye movements when humans tracking the arm in darkness are saccades (Dieter et al., 2014).

Second, new sensory modalities might be added to the proposed model. The proposed model is only a first step toward integrating cross-sensory prediction. While a straightforward extension might be to add additional inputs to the predictor network, consideration of the problem of adding new sensory modalities raises a number of intriguing questions. For example, which sensory inputs should be predicted from which others? Here we have considered prediction in only one direction. Another question is how to deal with the different possible combinations of sensory cues that might be available.

Third, the arm trajectory generation could be made more biologically realistic, e.g., through the use of dynamical movement primitives for trajectory generation (Schaal, 2006), or through the use of goal babbling to choose the via points (von Hofsten, 2004). In this work, we used random babbling and trajectory generation for its simplicity. The use of dynamic movement primitives would alter the statistics of the image motion induced by the hand, which might change the smooth pursuit performance, e.g., the final steady state error in Figure 6 or the shape of the frequency response curves in Figure 9. The use of goal babbling might improve the speed of learning (Baranes and Oudeyer, 2013). These would be interesting extensions of the model to investigate. However, we do not expect that their incorporation to change the main qualitative findings we report here, e.g., the ordering of the degradation in performance and proprioceptive or visual cues are removed.

Finally, the model might be integrated into a more comprehensive framework for hand-eye coordination that would include other tasks, such as reaching. In a sense, reaching is the inverse of the problem studied here. Our model generates commands to change eye gaze based on visual information and arm motion. Visually guided reaching involves the generation of an arm motion command to move the end effector to a visual target. The required mappings, e.g., between gaze direction and/or visual target location and end effector motion are often learned using a motor babbling process similar to that used here, where the end effector is tracked as the arm moves (Burger et al., 2018). In other cases, gaze is controlled to bring the target object or end effector to the image center (Huelse et al., 2010; Jamone et al., 2012; Savastano and Nolfi, 2013). In these works, the problem of tracking the end effector/target was simplified by attaching a marker to the end effector/target. Since our model does not require explicit markers, it might be used to relax some of the assumptions made by prior work. Because the location of the eye gaze drifts on the palm area (Figure 10), gaze direction does not directly correspond to a specific position on the end effector in trajectories generated by the model. However, the model could be used to generate data to learn an approximate mapping between gaze direction and end effector position.

## Author contributions

All authors participated in designing experiments and writing the paper. LW conducted the experiments.

### Conflict of interest statement

The authors declare that the research was conducted in the absence of any commercial or financial relationships that could be construed as a potential conflict of interest.

## Footnotes

^1^The video is available online at https://youtu.be/PPb7KwWefBI

^2^The video is available online at https://youtu.be/RsGTmbb0cf0
